# Functional Significance of the Adcy10-Dependent Intracellular cAMP Compartments

**DOI:** 10.3390/jcdd5020029

**Published:** 2018-05-11

**Authors:** Sofya Pozdniakova, Yury Ladilov

**Affiliations:** 1 Institute of Gender in Medicine, Center for Cardiovascular Research, Charite, 10115 Berlin, Germany; yury.ladilov@charite.de; 2 DZHK (German Center for Cardiovascular Research), Berlin Partner Site, 10115 Berlin, Germany

**Keywords:** adcy10, cAMP, phosphodiesterase, compartmentalization

## Abstract

Mounting evidence confirms the compartmentalized structure of evolutionarily conserved 3′–5′-cyclic adenosine monophosphate (cAMP) signaling, which allows for simultaneous participation in a wide variety of physiological functions and ensures specificity, selectivity and signal strength. One important player in cAMP signaling is soluble adenylyl cyclase (sAC). The intracellular localization of sAC allows for the formation of unique intracellular cAMP microdomains that control various physiological and pathological processes. This review is focused on the functional role of sAC-produced cAMP. In particular, we examine the role of sAC-cAMP in different cellular compartments, such as cytosol, nucleus and mitochondria.

## 1. Introduction

Even though 3′–5′-cyclic adenosine monophosphate (cAMP) was discovered more than half a century ago, it still remains an object of scientific interest. cAMP signaling plays an important role in a wide variety of physiological processes: transcription regulation [1,2], metabolism [3,4], cell migration [5,6], mitochondrial homeostasis [7,8,9,10,11] (reviewed in Reference [12]), as well as cell proliferation [13] (reviewed in Reference [14]) and cell death [15] (reviewed in Reference [16]). The importance of cAMP signaling is underlined by the fact that this pathway is evolutionarily conserved and can be found in all species from microorganisms to mammals [17,18,19].

There are two main sources of cAMP in the cell: Transmembrane (tmAC) and intracellularly localized soluble adenylyl cyclases (sAC). In mammalian cells, nine genes encode tmAC and one gene encodes sAC. The structural organization of tmAC is common for all members of this subfamily (9 tmAC) and the activity of tmAC is controlled by hormones and neurotransmitters [20,21]. Two important properties characterize the principal difference between tmAC and sAC: First, Gs, Gi, Gαi/o, Gßγ and Gq proteins regulate tmAC activity [22,23], whereas sAC activity is regulated by bicarbonate [24]; second, tmAC’s localization is restricted to the plasma membrane, while sAC is widely distributed within the cell and organelles [25]. The distinct spatial distribution of the two main cAMP sources leads to the formation of multiple intracellular cAMP compartments, thereby enabling the specificity and selectivity of cAMP signaling.

The specificity of cAMP signaling is further achieved through the restriction of cAMP diffusion due to physical barriers, i.e., mitochondria [26], and phosphodiesterases (PDEs) [27,28,29]. Therefore, with the exception of a rare internalization of tmAC [30], cAMP produced by tmAC under physiological conditions is mainly localized close to the plasma membrane. In contrast, sAC builds cAMP pools within various cellular compartments, e.g., cytosol, mitochondria, nucleus or the subplasmalemmal compartment [31,32,33]. In this review, we focus on sAC-dependent cAMP signaling, with a particular focus on its role in mitochondrial biology.

## 2. Structure and Regulation of sAC Activity

### 2.1. Structure

Mammalian sAC shows structural and functional similarities with cyanobacterial sAC [24], which argues for a bacterial origin of mammalian sAC that has been strongly conserved throughout the process of evolution [34]. The structure of the sAC catalytic core has a typical Class III pseudo-heterodimer arrangement of structurally similar C_1_ (residues 34–219) and C_2_ (residues 288–463) domains positioned at the N-terminus and connected by a linker [35]. The C-terminal region of sAC starts with a small motive, mediating auto-inhibitory effect [36], that most likely acts together with the neighboring putative NTPase domain [37]. Additionally, the C-terminal region contains a heme-binding domain that can bind nitric oxide (NO), carbon monoxide, and other potential gaseous signaling molecules [38]. Active cyclase is a heterodimer of two catalytic domains [19]. sAC is encoded by a single functional sAC gene in the human genome (*ADCY10*), comprising of 33 exons covering approximately 104 kb of genomic DNA [39,40]. sAC mRNA undergoes extensive alternative splicing which leads to smaller splice variants [41]. In mammalian cells the predominant isoform is a 50 kDa truncated sAC (sAC_t_) which is categorized as a splice variant of the full-length enzyme. sAC_t_ is restricted to the N-terminal part of the full-length protein covering C_1_ and C_2_ [42]. sAC_t_ shows a higher activity than the full-length enzyme, as the activity of the latter is suppressed by the small auto-inhibitory module at the C-terminal [36]. More splice sAC variants have also been identified in human somatic tissue. These isoforms predominantly consist of C_2_ domain and require a partner protein to become active due to a missing or incomplete C_1_ domain [19,43].

### 2.2. Posttranslational Regulation of sAC

sAC is insensitive to heterotrimeric G-protein regulation due to a missing or modified G_sα_ and G_ßγ_ binding region, which is important for the activation of tmAC [44]. A recent study performed by Hebert-Chatelain et al., however, challenged this paradigm of sAC insensitivity to G proteins. The authors demonstrated that the activation of mitochondrial G_αi_ proteins through cannabinoid receptors inhibits mitochondrial sAC [45]. However, the authors investigated the role of sAC applying the sAC inhibitor KH7, which may have also led to sAC-independent effects on the mitochondria [9,46]. The results could also be explained by an indirect downregulation of sAC activity.

sAC activity requires divalent metal cations in the catalytic active site of the enzyme in order to coordinate the binding and cyclizing of ATP. sAC is most active in the presence of Mn^2+^, however it is not clear whether the physiological intracellular Mn^2+^ concentration would support sAC activity [47]. Mg^2+^ and Ca^2+^ concentrations within the expected intracellular range 1–10 mmol/L for Mg^2+^ and 2–1200 nmol/L for Ca^2+^ make significant contributions to the regulation of sAC activity [43]. Furthermore, sAC serves as an intracellular ATP sensor because its activity is dependent on physiological changes in ATP concentrations. When the ATP level is reduced, sAC shows decreased activity due to substrate limitation [48].

A unique property of sAC is its activation through bicarbonate binding, which makes sAC the only protein with enzymatic activity regulated by bicarbonate. Bicarbonate directly binds to and activates sAC in a pH-independent manner [24]. The EC_50_ for the bicarbonate stimulation of mammalian sAC is within the 10–25 mmol/L range, which is appropriate for sensing physiological bicarbonate levels of 2–25 mmol/L [24]. It is also worth mentioning that sAC activity increases synergistically in the presence of bicarbonate and Ca^2+^ [43,49].

### 2.3. Pharmacological Regulation of sAC

sAC is involved in a wide variety of physiological processes, including metabolism, proliferation, apoptosis, differentiation, migration development, ion transport, pH regulation and gene expression (reviewed in [16,47]). It is also involved in different pathologies such as hyperproliferative skin disease, hypercalciuria, type 2 diabetes glaucoma and prostate cancer [40,50,51,52,53,54,55]. Therefore, the pharmacological inhibition or activation of sAC may be considered for the treatment of the pathologies and the maintenance of the physiological processes mentioned above. Although the search for potential sAC activators remains unsuccessful, several inhibitors have been discovered. Catechol estrogens (CEs) are physiologically occurring steroid derivatives that can inhibit mammalian AC enzymes. 2-hydroxy estradiol (2-CE) and 4-hydroxy estradiol (4-CE) inhibit purified mammalian sAC (IC_50_ 2–8 μmol/L) as well as some purified tmAC isoforms with comparable potency [44,56]. CEs are postulated to be non-competitive inhibitors of AC that bind to a pocket near the enzyme’s active site [57].

Another potent sAC inhibitor is (*E*)-2-(1*H*-Benzo[d]imidazol-2-ylthio)-*N*′-(5-bromo-2-hydroxybenzylidene) propanehydrazide (KH7) (IC_50_~3 μmol/L) [58]. KH7 shows good membrane permeability and has no significant effect on tmACs, GC or PDEs up to a concentration of 100 µmol/L [56]. KH7 has been used as a pharmacological tool in a large number of studies and seems to be a promising compound for drug development. Unfortunately, KH7 exhibits an intrinsic fluorescence and is therefore of limited use when studies involve fluorescence-based live cell cAMP sensors, according to our own observations and research [9]. In addition, KH7 leads to mitochondrial uncoupling in a sAC-independent manner [9,46]. Therefore, KH7 use should be restricted to short-term assays and the results should be interpreted carefully.

Recently, LRE1—an improved sAC-specific inhibitor—has been identified [46]. LRE1 inhibits sAC by occupying the bicarbonate binding site. LRE1 neither exhibits cell toxicity nor results in uncoupling of isolated brain mitochondria [46]. In our experiments, we have not observed any interference between LRE1 and fluorescence, which allows the compound to be used in live cell imaging.

## 3. Functional Role of sAC in Different Cellular Compartments

sAC-generated cAMP is involved in the regulation of multiple cellular functions as it is generated locally within particular microdomains containing cAMP effectors (PKA, EPAC, cyclic nucleotide-gated ion channels and Popeye domain-containing proteins [59,60,61,62]), scaffolding proteins (A-kinase anchoring proteins, AKAPs) and a subset of PDEs, that degrade cAMP, and thus suppress cAMP diffusion [28,29,63]. AKAPs form the complexes of cAMP and its downstream targets, and bind these complexes to particular subcellular compartments [22]. Tight spatiotemporal regulation of cAMP dynamics inside discrete signaling compartments provides specific responses to diverse stimuli at certain locations and avoids unregulated cross-communication between microdomains.

**Figure 1:**
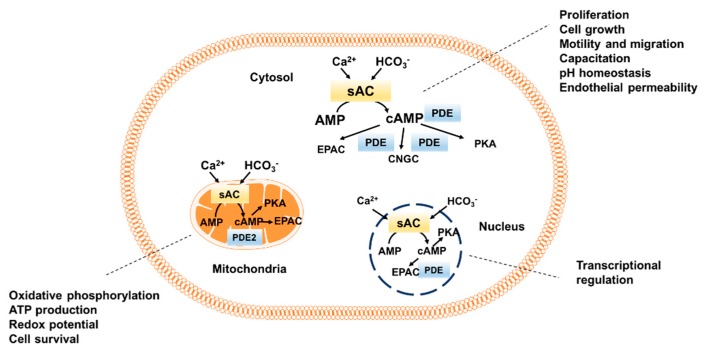
Intracellular distribution of sAC-dependent cAMP pool. sAC, soluble adenylyl cyclase; PKA, protein kinase A, EPAC, exchange protein directly activated by cAMP; CNGC, cyclic nucleotide gated channels; PDE, phosphodiesterase.

Mammalian sAC is distributed over different compartments throughout the cell: the cytosol, nucleus, plasma membrane and mitochondria [25,64,65,66,67,68]. Although numerous cellular functions have been attributed to the activity of sAC, the functional significance of sAC in particular compartments is still in need of clarification. Therefore, in this review, the functional significance of different sAC domains will be described according to the sAC subcellular localization (Figure 1).

### 3.1. Role of sAC-Dependent cAMP Signaling in Microtubules and Centrioles

It has been suggested that sAC both co-localizes with microtubules and centrioles, while also playing a role in mitosis and cytokinesis [25]. During prophase, sAC is dispersed from the nucleus. In metaphase and anaphase, it accumulates at the mitotic poles and spindle fibers. During cytokinesis, sAC is localized in the midbody. In the centrioles, the main pathway that promotes the phosphorylation cascade is PKA-dependent, whereas in the microtubules it is EPAC-dependent [25].

### 3.2. Role of Cytosolic/Nuclear sAC-Dependent cAMP Signaling

#### 3.2.1. Proliferation and Cell Growth

Cytosolic sAC makes a significant contribution to the regulation of cell growth, particularly in hyperplasia [16]. In prostate carcinoma tissue and cells (LNCaP, PC3), sAC was shown to be overexpressed and the suppression of sAC activity significantly reduced the proliferation rate [53]. A subsequent analysis of the underlying cellular mechanisms revealed the role of the EPAC/Rap1/B-Raf axis in the sAC-dependent regulation of cell growth. Inhibiting sAC down regulates cyclin B_1_ and cyclin-dependent kinase 1, which are the key proteins involved in the G2/M transition. Thus, sAC suppression causes cell cycle arrest in the G2 phase [53]. In another tumor cell line (PC12), nerve growth factor stimulation via sAC was shown to induce cAMP elevation, which, in turn, promoted the activation of Rap1 [69]. This mechanism is considered to be implicated in the process of brain-derived neurotrophic factor-mediated axonal guidance. A study performed in breast cancer cells postulated that sAC in the EPAC-Rap1 dependent mechanism is involved in a metabolic switch, thereby favoring the development of malignant progression [70].

sAC also plays a role in non-proliferative cell growth, i.e., hypertrophy. It is expressed in embryonic neurons and generates cAMP in response to netrin-1, a member of the laminin-related secreted proteins family, thus affecting axon outgrowth [71]. Moreover, retinal ganglion cell survival and axon growth is regulated by Ca^2+^-dependent cAMP-PKA signaling [64]. Our recent study revealed a novel role for sAC in cardiac hypertrophy induced by either β-adrenergic stimulation or pressure overload [72]. B-Raf’s involvement in sAC-dependent hypertrophy was also demonstrated in that study.

#### 3.2.2. Motility

sAC plays a central role in sperm physiology [58,73]. During one of the first definable events in capacitation, Ca^2+^ and bicarbonate enter into sperm and activate sAC to produce cAMP. This promotes an asymmetrical flagellar beat frequency and results in vigorous forward sperm motility [47]. In keeping with this role of sAC in sperm motility, male sAC knockout mice show an infertility phenotype [74]. Though sAC’s role in cell motility was initially exclusively considered for sperm, a recent report suggested that sAC is also involved in the regulation of leukocyte trans-endothelial migration through the CD99 [75]. CD99 and sAC are co-localized in a signaling complex with ezrin and PKA. The stimulation of CD99 promotes the sAC-PKA pathway that activates membrane trafficking from the lateral border recycling compartment to sites of trans-endothelial migration, facilitating the passage of leukocytes across the endothelium [75].

#### 3.2.3. pH Homeostasis

sAC plays an important role in the regulation of pH homeostasis [76,77]. In epididymal clear cells and in kidney intercalated cells, sAC-produced cAMP promotes the translocation of the vacuolar proton pumping ATPase (V-ATPase) to the acid-secreting surface in a PKA-dependent manner [78,79]. The apical translocation of V-ATPase, associated with the protein activation, plays an important role in the regulation of pH homeostasis and extracellular acidification/alkalinization. The maintenance of acid/base balance is important for the regulation of acids in the body. V-ATPase dysfunction is one of the factor that leads to renal distal tubular acidosis, the formation of kidney stones and proteinuria [80].

Recently, sAC’s control of the endosomal-lysosomal acidification has been shown to function in a PKA-dependent manner. The absence of sAC disrupts V-ATPase localization at the lysosomal membrane which is rescued by treatment with membrane-permeable cAMP [81]. It is interesting to note that a disturbance in lysosomal acidification through sAC knockout leads to an impaired autophagic degradative system.

#### 3.2.4. Transcriptional Regulation

An increasing number of reports argue for the essential role of sAC in regulating the transcriptional activity of the cell. Indeed, sAC has been identified as a unique source of cAMP in the nucleus that in PKA-dependent manner regulates CREB activity [68]. sAC, in a PKA-dependent manner, is especially involved in corticotropin-releasing hormone-mediated CREB phosphorylation and c-fos (endogenous CREB target) induction in hippocampal neuronal cells [82]. A recent study demonstrated that sAC contributes to the regulation of CREB-mediated Na^+^/K^+^-ATPase expression in the vascular endothelium and is an important regulator of endothelial stiffness [83,84]. Besides promoting CREB activity, sAC also regulates several other transcription factors. For example, sAC supports hypercapnia-accelerated adipogenesis via the activation of pro-adipogenic transcription factors, such as CREB, CCAAT/enhancer binding protein ß and proliferator-activated receptor γ [85]. Similarly, sAC-PKA-dependent phosphorylation, and thus the activation of transcription factor 4, is required for brain development [86].

#### 3.2.5. CFTR Regulation

The Cystic Fibrosis Transmembrane Conductance Regulator (CFTR) is a chloride channel, primarily localized in the apical membrane of secretory epithelial cells. Mutations in the CFTR lead to the development of cystic fibrosis [87]. In cultured human airway epithelial cells, it has been found that sAC, activated by bicarbonate, modulates CFTR function in a PKA-dependent manner. The inhibition of sAC attenuated bicarbonate-stimulated CFTR activity [88]. Further studies have demonstrated that CFTR is involved in bicarbonate entry into granulosa cells, which further promotes the nuclear cAMP-PKA-CREB axis [89]. CFTR is involved in triggering sperm capacitation, as CFTR promotes bicarbonate secretion by the endometrium [90] which, in turn, activates sAC in sperm, increases cAMP production, and then activates PKA and the cyclic nucleotide gate cation channels [91,92]. Moreover, CFTR via the sAC-cAMP-PKA pathway has been shown to promote embryo development through the suppression of p53-dependent development arrest [93]. Taken together, the CFTR-sAC axis seems to play an important role in reproductive processes [94].

#### 3.2.6. Na^+^/K^+^-ATPas Endocytosis

In alveolar epithelial cells, a high CO_2_ concentration promotes the sAC-cAMP axis, which in turn induces a PKA-dependent phosphorylation of α-adducin, a component of the actin cytoskeleton, resulting in Na^+^/K^+^-ATPase endocytosis [95]. In the vascular endothelium, the role that the sAC-cAMP axis plays in Na^+^/K^+^-ATPase regulation has been demonstrated, as the inhibition of sAC (KH7 and interfering RNA) significantly decreases the mRNA and protein levels of Na^+^/K^+^-ATPase [84]. A recent study confirmed that the sAC-dependent regulation of Na^+^/K^+^-ATPase in the vascular endothelium plays an important role in endothelial stiffness [83].

#### 3.2.7. Endothelial Permeability

The importance of the intracellular distribution of cAMP for endothelial barrier function has been demonstrated, because the stimulation of plasma membrane and cytosolic cAMP pools exerts the opposite effects [96,97].

A recent study suggested that sAC has a protective effect on endothelial barrier function under inflammatory and hypoxic conditions [98]. In this study, the bicarbonate-mediated activation of sAC elevated cellular cAMP levels was followed by PKA and EPAC activation, which led to the inhibition of RhoA/Rock signaling and the translocation of VE-cadherin at cell–cell junctions. Moreover, sAC activation abrogated thrombin and hypoxia/reoxygenation-induced endothelial cells hyperpermeability. Pharmacological inhibition or knockdown of sAC worsened the thrombin-induced endothelial hyperpermeability suggesting that basal sAC activity is required for the maintenance of the endothelial barrier function under inflammatory conditions.

### 3.3. Role of Mitochondrial sAC-Dependent cAMP Signaling

#### 3.3.1. Extra-Mitochondrial sAC

According to the current view on mitochondrial cAMP signaling, two main cores that contain distinct cAMP signaling pathways—the extra-mitochondrial sAC (outer mitochondrial membrane (OMM)) and intra-mitochondrial (the mitochondrial matrix)—can be distinguished [99]. The specificity of cAMP in OMM is mainly achieved through PKA tethering to OMM by several AKAPs, which allows multiple processes to be carried out, including mitochondrial protein import, autophagy, mitophagy, mitochondrial fission and fusion, and apoptosis [99]. Our recent study defined the role that sAC plays in regulating mitochondrial biogenesis and mitophagy [100].

It has been demonstrated that the cytosolic pool of cAMP generated by sAC is also involved in controlling mitochondrial apoptosis. Under stress conditions, the translocation of cytosolic sAC to the mitochondria leads to a selective activation of PKA, followed by phosphorylation and binding of the pro-apoptotic protein Bax to mitochondria and the release of cytochrome c in coronary endothelial cells, cardiomyocytes and aortic smooth muscle cells [15,101,102]. Furthermore, the overexpression of cytosolic sAC, but not intra-mitochondrial sAC, promotes the activation of the mitochondrial pathway of apoptosis under oxysterol treatment [102].

#### 3.3.2. Intra-Mitochondrial sAC

An increasing amount of evidence suggests that intra-mitochondrial cAMP/PKA signaling is present in mammals [8,45,103] and yeast [104]. Although transmembrane adenylyl cyclase was initially assumed to be a source of mitochondrial cAMP [105], a recent study [9] reconsidered this paradigm and demonstrated that cytosolic cAMP cannot permeate the inner mitochondrial membrane and a mitochondria-localized cAMP source, i.e., sAC, is required [99]. In a recent study [106] we confirmed the previously published findings [9] that activating plasmalemmal adenylyl cyclase with forskolin leads to a rapid elevation of cytosolic cAMP, but does not affect cAMP concentration in mitochondria. It is worth noting that we [106], as well as other authors [9,107,108], have all observed a rapid increase in intra-mitochondrial cAMP under sAC stimulation with bicarbonate.

Bicarbonate and Ca^2+^ stimulation of mitochondrial sAC may couple the activity of the TCA cycle—the main source of CO_2_/bicarbonate in the cell—and alterations in the intra-mitochondrial Ca^2+^ concentration to the OXPHOS activity [9]. Indeed, the seminal studies of Acin-Perez et al. demonstrated that cAMP produced in the mitochondrial matrix promotes cytochrome c oxidase activity via a PKA-dependent phosphorylation of cytochrome c oxidase subunit IV [8,109]. Knockout of sAC in fibroblasts causes a decline in OXPHOS activity that is compensated with elevated OXPHOS expression, whereas restoring sAC expression in the mitochondrial matrix rescues OXPHOS activity [110]. Similar results (regulation of OXPHOS activity via cAMP-PKA axis) were obtained in yeast, where the inhibition of sAC caused a decline in respiration and OXPHOS activity [104]. Furthermore, in a study on human fibroblasts, the inhibition of sAC depressed complex I activity was rescued by adding a membrane-permeable cAMP analog [111].

The role of intra-mitochondrial sAC in the regulation of memory processing was recently demonstrated [45]. The authors suggested that the activation of mitochondrial localized type-1 cannabinoid receptors (mtCB_1_) decreases mitochondrial cAMP, complex I activity, mitochondrial respiration and cellular ATP content in hippocampal cell culture. In their study bicarbonate stimulation fully reversed the effect of mtCB_1_-receptor activation and eliminated the cannabinoid-induced reduction in respiration. The study also confirmed that the modulation of brain mitochondrial respiration occurs through the PKA-dependent phosphorylation of complex I subunit NDUFS2 [45].

In addition to the post-translational regulation of OXPHOS activity via PKA-dependent phosphorylation, [8] it has also been suggested that sAC has an effect on the turnover of OXPHOS proteins. Indeed, intra-mitochondrial cAMP prevents the digestion of nuclear-encoded subunits of complex I by mitochondrial proteases and supports its NADH-ubiquinone oxidoreductase activity [111].

Aside from the above-mentioned studies, several other reports have demonstrated the presence of functional PKA in the mitochondrial matrix [112,113]. In a notable study that applied a PKA-sensing system with a robust dynamic range, Agnes et al. [113] characterized the compartmentalized location of PKA activity as being in bovine heart mitochondria. The experimentally determined PKA activity ratio—79:8:13 in mitochondrial matrix/intermembrane space/outer membrane respectively—provided evidence that the major PKA activity is located in the mitochondrial matrix. In agreement with that study, Sardanelli et al. [112], applying densitometric immunoblot analysis and activity assays, concluded that the majority (~90%) of mitochondrial PKA is localized in the inner mitochondrial compartment. Nevertheless, this issue of PKA localization is still a matter of debate [114]. Indeed, applying FRET-based analysis of PKA activity Lefkimmiatis et al. found no evidence of PKA activity in the mitochondrial matrix [115]. In addition, it was demonstrated that calcium-induced cardiac mitochondrial respiration is PKA independent [116]. This obvious discrepancy may be due to differences in the methods used in the analysis of PKA activity or cell models (reviewed in Valsecchi et al. [114]). In fact, the absence of PKA activity in Lefkimmiatis’s study may be due to the use of predominantly glycolytic cell lines, i.e., HeLa and HEK cells. In addition, in many studies PKA activity was examined through treatment with H89, which is an unspecific PKA inhibitor and may lead to numerous side effects.

Though PKA has long been considered the most active kinase in the matrix and the main effector of intra-mitochondrial cAMP [12], another cAMP downstream target involved in the regulation of mitochondrial function—EPAC—has also been described [117]. The mitochondrial sAC-cAMP-EPAC pathway regulates coupling efficiency and the structural organization of F_0_F_1_ATP synthase in mammalian mitochondria [118]. In a recent study, Wang et al. [107] demonstrated a down-regulation of sAC in an animal model of heart failure, which was accompanied by a reduced resistance to Ca^2+^ overload in cardiac mitochondria. The authors underlined the inhibitory effect of the sAC/cAMP/Epac1 axis on the Ca^2+^ overload-induced opening of mitochondrial permeability pore transition [107]. In contrast, a study by Fazal et al. [119] postulated that activation of the mitochondrial sAC-cAMP-EPAC axis stimulates the mitochondrial Ca^2+^ entry, the opening of mitochondrial permeability pore transition and cell death.

#### 3.3.3. Intra-Mitochondrial PDE2

In addition to the sAC, PDEs also contribute to the intra-mitochondrial cAMP level. PDE2A has been found to be a predominant intra-mitochondrial isoform [103]. This PDE is activated by cGMP that enables a negative cGMP-cAMP cross-talk [103,120]. A study performed with mitochondria isolated from mouse brains suggested that PDE2A, and the PDE2A2 isoform in particular, is localized in the mitochondrial matrix—due to the mitochondrial targeted sequence at N terminus of PDE2A2—where it regulates the activity of the mitochondrial respiratory chain [103]. Applying the super-resolution stimulated emission depletion microscopy in neonatal rat ventricular myocytes, Monterisi et al. revealed the localization of PDE2A outside of the mitochondrial matrix, particularly at the outer or inner mitochondrial membrane, where it regulates mitochondrial morphology, mitochondrial membrane potential and cell death via sAC-independent mechanisms [121]. Further investigation is required to clarify the localization and activity of PDE2 in mitochondria.

Since the PDE2 is activated by cGMP, it is tempting to speculate that an activation of NO signaling may lead to the activation of mitochondrial PDE2. In fact, our new report demonstrated a decline in mitochondrial cAMP concentration after NO signaling activation, either by NO donor or estradiol, in a PDE2- and sGC-dependent manner [106]. It is worth nothing that the localization of sGC in mitochondria was confirmed by western blot analysis. The reduction of mitochondrial cAMP level was accompanied by a decline in mitochondrial COX activity in a PDE2-dependent manner [106]. These data are in agreement with a previous report that demonstrated that the inhibition of PDE2A with BAY60-7550 increases oxygen consumption and ATP production in isolated mitochondria [103].

To prove whether the beneficial effect of PDE2 inhibition may be translated to cardiac pathology, adult rat cardiomyocytes were challenged metabolically with cyanide followed by a recovery phase. Inhibition of PDE2A with BAY60-7550 significantly improved cell viability [122]. In alignment with these results, a recent report suggested that PDE2 inhibition has a protective effect in a brain ischemia/reperfusion model, although it was delayed rather than acute effects of reperfusion that were analyzed [123]. Similarly, an inhibition of matrix localized PDE2A with BAY60-7550 reduced the uncoupled respiration rate and increased cytochrome c oxidase activity in septic mice [124].

### 3.4. Importance of sAC in the Cardiovascular System

The role of cAMP in the regulation of numerous physiological and pathological processes in the heart is well known [125,126,127]. Nevertheless, knowledge about the role of sAC in the cardiovascular system is limited. A seminal study by Sayner et al. [97] showed sAC’s regulation of endothelial barrier function. We have also demonstrated that sAC plays a role in cardiovascular apoptosis [15,101]. The importance of sAC in cardiac pathology, like heart failure, has recently been suggested by Wang et al. [107]. The authors revealed a dramatic downregulation of sAC in mitochondrial fraction isolated from rat hearts at the late phase of cardiomyopathy and linked it to the reduced Ca^2+^ resistance of mitochondria. Our recent study presented further evidence of the importance of sAC in cardiac hypotrophy induced by isoprenaline (isolated cardiomyocytes) or pressure overload (sAC-knockout mice) [72].

## 4. Conclusions

cAMP signaling plays a fundamental role in controlling numerous cellular functions. The system is complex and has a well-organized spatiotemporal structure. Different mechanisms are involved in the compartmentalized structure of cAMP within the cell, including phosphodiesterases, tmAC- and sAC-dependent cAMP sources. The discovery of sAC as an alternative, intracellular source of cAMP significantly expands our knowledge of the spatial compartmentalization of cAMP signaling. The multifunctional role of sAC in the regulation of mitochondrial function and transcriptional activity in the cells, together with other functions described in this review, shows how important this cyclase is for cellular and organismal homeostasis and health. In this light, an in-depth understanding of sAC biology may contribute significantly to the prevention, prediction and treatment of several pathologies. The appearance of recent data describing the role of sAC in cardiovascular physiology [9] and pathology [11,15,72,102,107,119,124] is not surprising, especially considering the fundamental role that cAMP signaling plays in the regulation of heart function.

## Abbreviations

cAMP	3′–5′-cyclic adenosine monophosphate
sAC	soluble adenylyl cyclase
tmAC	transmembrane adenylyl cyclase
PDEs	phosphodiesterases
PKA	protein kinase A
EPAC	exchange protein directly activated by cAMP
CREB	cAMP-response element binding protein
CE	Catechol estrogens
GC	guanylyl cyclase
AKAP	A-kinase anchoring proteins
CFTR	cystic fibrosis transmembrane conductance regulator
V-ATPase	vacuolar H^+^-ATPase
IMS	intra-mitochondrial space
OMM	outer mitochondrial membrane
IMM	inner mitochondrial membrane
NTPase	nucleoside-triphosphatase
OXPHOS	oxidative phosphorylation
